# Beam characteristics of the first clinical 360° rotational single gantry room scanning pencil beam proton treatment system and comparisons against a multi‐room system

**DOI:** 10.1002/acm2.12984

**Published:** 2020-08-13

**Authors:** Charles Shang, Grant Evans, Mushfiqur Rahman, Liyong Lin

**Affiliations:** ^1^ South Florida Proton Therapy Institute Delray Beach FL USA; ^2^ Emory Proton Therapy Center Atlanta GA USA

**Keywords:** commissioning, pencil beam scanning, protons, treatment planning system

## Abstract

**Purpose:**

The purpose of this study was to present the proton beam characteristics of the first clinical single‐room ProBeam Compact™ proton therapy system (SRPT) and comparison against multi‐room ProBeam™ system (MRPT).

**Materials and Methods:**

A newly designed SRPT with proton beam energies ranging from 70 to 220 MeV was commissioned in late 2019. Integrated depth doses (IDDs) were scanned using 81.6 mm diameter Bragg peak chambers and normalized by outputs at 15 mm WET and 1.1 RBE offset, following the methodology of TRS 398. The in‐air beam spot profiles were acquired by a planar scintillation device, respectively, at ISO, upper and down streams, fitted with single Gaussian distribution for beam modeling in Eclipse v15.6. The field size effect was adjusted for the best overall accuracy of clinically relevant field QAs. The halo effects at near surface were quantified by a pinpoint ionization chamber. Its major dosimetric characteristics were compared against MRPT comparable beam dataset.

**Results:**

Contrast to MRPT, an increased proton straggling in the Bragg peak region was found with widened beam distal falloffs and elevated proximal transmission dose values. Integrated depth doses showed 0.105–0.221 MeV (energy sigma) or 0.30–0.94 mm broader Bragg peak widths (R_b80_–R_a80_) for 130 MeV or higher energy beams and up to 0.48–0.79 mm extended distal falloffs (R_b20_–R_b80_). Minor differences were identified in beam spot sizes, spot divergences, proton particles/MU, and field size output effects. High passing scores are reported for independent end‐to‐end dosimetry checks by IROC and for initial 108 field‐specific QAs at 3%/3 mm Gamma index with fields regardless with or without range shifters.

**Conclusions:**

The author highlighted the dosimetry differences in IDDs mainly caused by the shortened beam transport system of SRPT, for which new acceptance criteria were adapted. This report offers a unique reference for future commissioning, beam modeling, planning, and analysis of QA and clinical studies.

## 1 INTRODUCTION

The first 360° rotational single gantry room scanning pencil beam proton treatment system (SRPT) — ProBeam Compact™ (Varian Medical, Palo Alto, CA) was implemented in a clinical setting in November 2019. This system mainly consists of (a) a superconducting cyclotron which accelerates and injects 250 MeV protons to the beamline; (b) open‐air energy selection system^1^ with carbon multi‐wedge technique for clinical beam energies ranging from 70 to 220 MeV; (c) shortened single‐room dedicated beam transport system removing two or three entrance bending magnets keeping a 45° and a 135° major bending magnets;^2^ (d) 360° rotating gantry equipped by two orthogonally arranged onboard kV imaging systems with CBCT capability; and (e) six‐dimensional (6D) robotic patient support system. Different from a conventional multi‐gantry ProBeam proton treatment system (MRPT), the newly designed components two and three contribute unique dosimetric characteristics of the scanning proton beams and there is no report of clinically relevant beam parameters, we attempt to fill this void here.

The principle beam dosimetric components of the commissioning typically comprised of integrated depth dose curves (IDDs), absolute dose calibration for given MUs (or dose output), in‐air beam spot size or profiles.^3^, ^4^, ^5^, ^6^ Other essential elements are virtual source position relative to ISO, beam spot accuracy and dose uniformity, field size factors (or halo effect of spot profile), MU linearity, mechanical accuracy, OBI and CBCT quality and accuracy, CT stoichiometric calibration, WET measurement for the range shifters, table support and inserts, as well as immobilization and physics accessories.^7^, ^8^, ^9^ However, only beam dosimetric characteristics will be discussed in this report.

## 2 MATERIALS AND METHODS

The commissioning of this ProBeam Compact™ was conducted in November 2019. IDD data were acquired by PTW 81.6 mm diameter Bragg peak plane–parallel chambers (Model 34070 primary and model 34080 reference) and a PTW 3D Water Scanning System MP30PL (Freiburg, Germany), using central axial downward (AP) proton beams in every 5 MeV energy intervals. Following the methodology recommended by TRS 398 report, the absolute dose output of each nominal monogenic beam was obtained at 15 mm depth in water aligned to the ISO using an ADCL calibrated PPC05 Markus parallel‐plate chamber (IBA Dosimetry). In addition, the known dosimetric output accuracy issues in PCS^3^, ^4^ were corrected with AcurosPT calculations for different test fields.^7^ To convert the measurements in the transmission beam region to radiation doses, 1.002 k_Q_ factor was used along with 1.1 RBE offset factor, which were then imported to an Eclipse v15.6 (Varian, Palo Alto, CA) for modeling the PCS and NUPO algorithms. Here, the low‐energy (85 to 70 MeV) dose outputs were slightly adjusted by using 0.995–0.990 for k_Q_ variations.^10^ The final IDD outputs particularly for AcurosPT were fine‐tuned for an optimal overall accuracy, based on the measurements of the calibrated ionization chamber two‐dimensional (2D) array on different testing field sizes and various patient plan‐specific QAs.^3^, ^4^ In this commissioning, AcurosPT algorithm was exclusively utilized for final planning dose computations.

The measurements of in‐air proton beam spots were accomplished using a 2D scintillator device, Logos XRV4000 Hawk beam profiler (Logos Systems Int'l, Scotts Valley, CA) at angles representing the average spot sizes of both X and Y axial dimensions. Then, the full width at half maximum (FWHM) of spots on both X and Y axes was determined using an in‐house Python program; the average spot sigma values were finally derived by the least‐square fitting of single Gaussian function for the current Eclipse modeling.

Validation of the modeling comprises the verifications of the computed IDDs and calculated dose in difference conditions and field sizes with both PCS and AcurosPT models^11^, ^12^, ^13^, ^14^, ^15^, ^16^; the overall accuracy of the AcurosPT‐computed treatment planning and dose delivery using realistic clinical plans as well as the end‐to‐end tests for different open beams and beams with different range shifters and air gaps.^17^, ^18^ The halo effects of the beams beyond the modeled profile were examined by comparison between the AcurosPT computed and measured doses with different field sizes and energies at near the surface.^8^ A PTW Semiflex 2.4 mm × 4.8 mm ion chamber (TN31021) was used for the measurements at ISO with 8 mm WET, matching the inherent buildup of PTW Octavius 1500XDR for the dose conversion. Octavius 2D ion chamber array, used for patient's specific QAs, already had an absolute dose calibration against an ADCL calibrated Farmer chamber.

The final confirmation for the overall commissioning accuracy includes the independent dosimetry checks by an outside physicist, IROC using different QA phantoms, and analysis of various clinical patient‐specific QA tests. The major dosimetric characteristics of the proton beams were compared against a recent MRPT dataset.

## 3 RESULTS

**Fig. 1:**
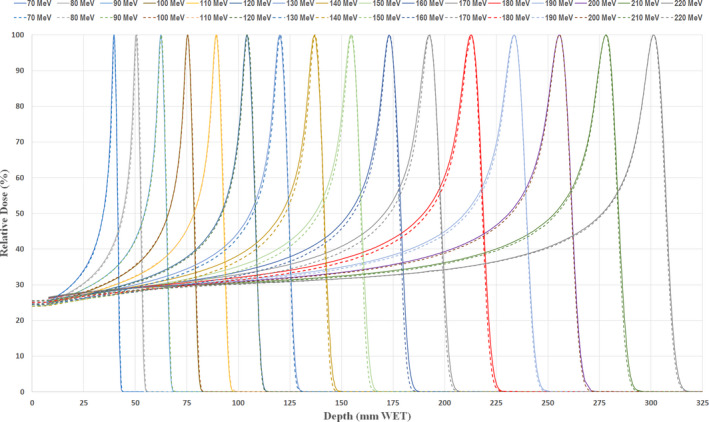
Comparison of the peak normalized integrated depth dose curves between SRMT (solid curves) and multi‐room ProBeam™ system (dotted curves) in every 10 MeV steps.

**Fig. 2:**
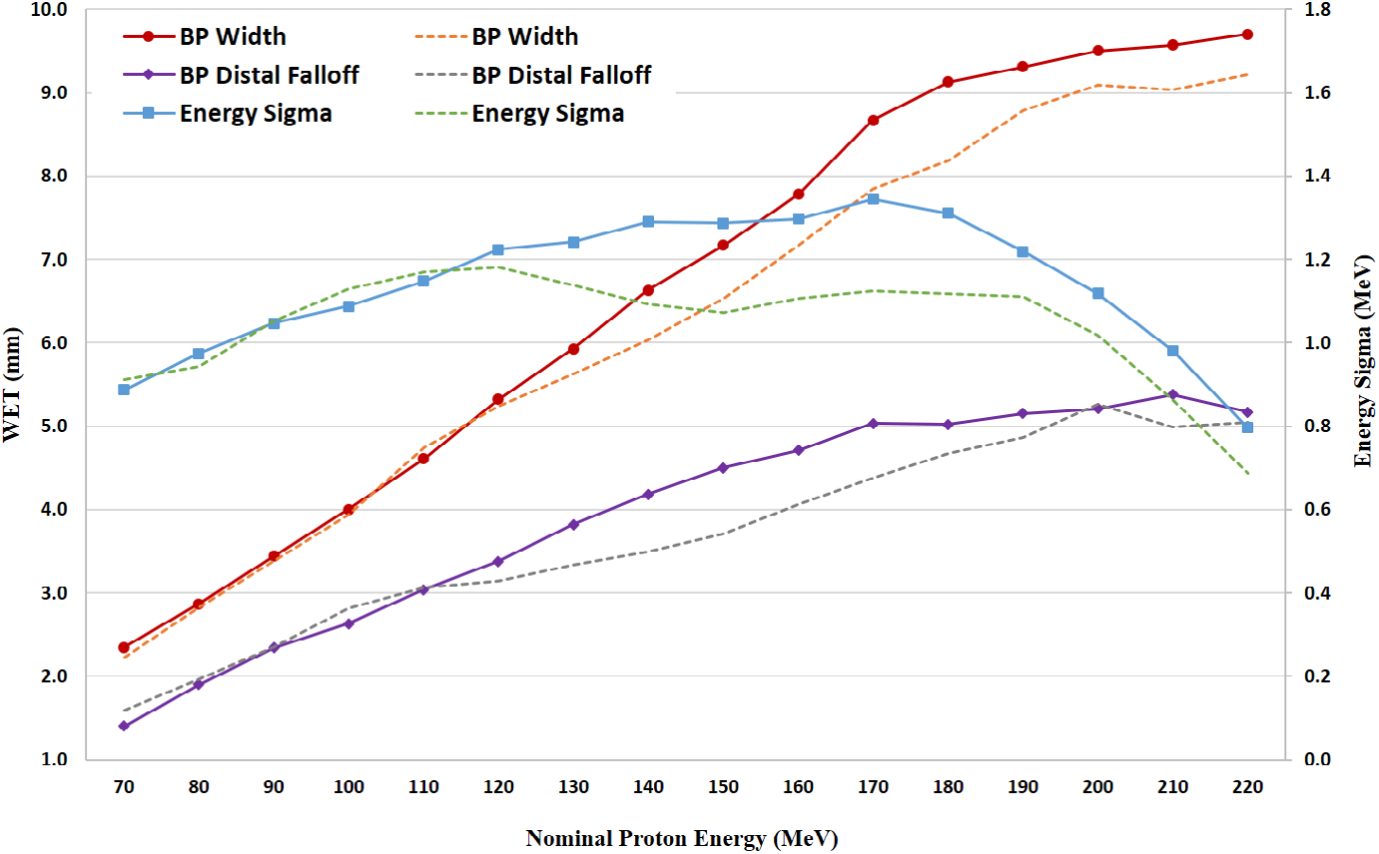
Comparison of Bragg peak (BP) dosimetry characteristics between single‐room ProBeam Compact™ proton therapy system (SRPT) (solid curves) and multi‐room ProBeam™ system (dotted curves) for BP widths, distal falloff, and the AcurosPT computed energy sigma, where larger differences shown in proton energies> 130 MeV.

The IDDs imported to Eclipse for PCS and NUPO modeling are incorporated with the absolute output doses and 1.1 RBE factor for each energy curve (see Fig. 1). Contract to MRPT data, the Bragg peaks from the ProBeam Compact™ have shown larger proton straggling with widened Bragg peaks and elevated proximal depth dose, more dominantly for the midrange of energies. The detailed differences of Bragg peak dosimetry characteristics are further illustrated in Fig. 2, where IDDs from SRPT show 0.105–0.221 MeV (energy sigma) or 0.30–0.94 mm increase in Bragg peak width (R_b80_–R_a80_) for energies of 130 MeV or greater, peaked at 180 MeV. In addition, about 0.48–0.79 mm extended distal falloffs are noticed in energies between 130 and 170 MeV, which coincide with increased energy sigma in AcurosPT modeling.

**Fig. 3:**
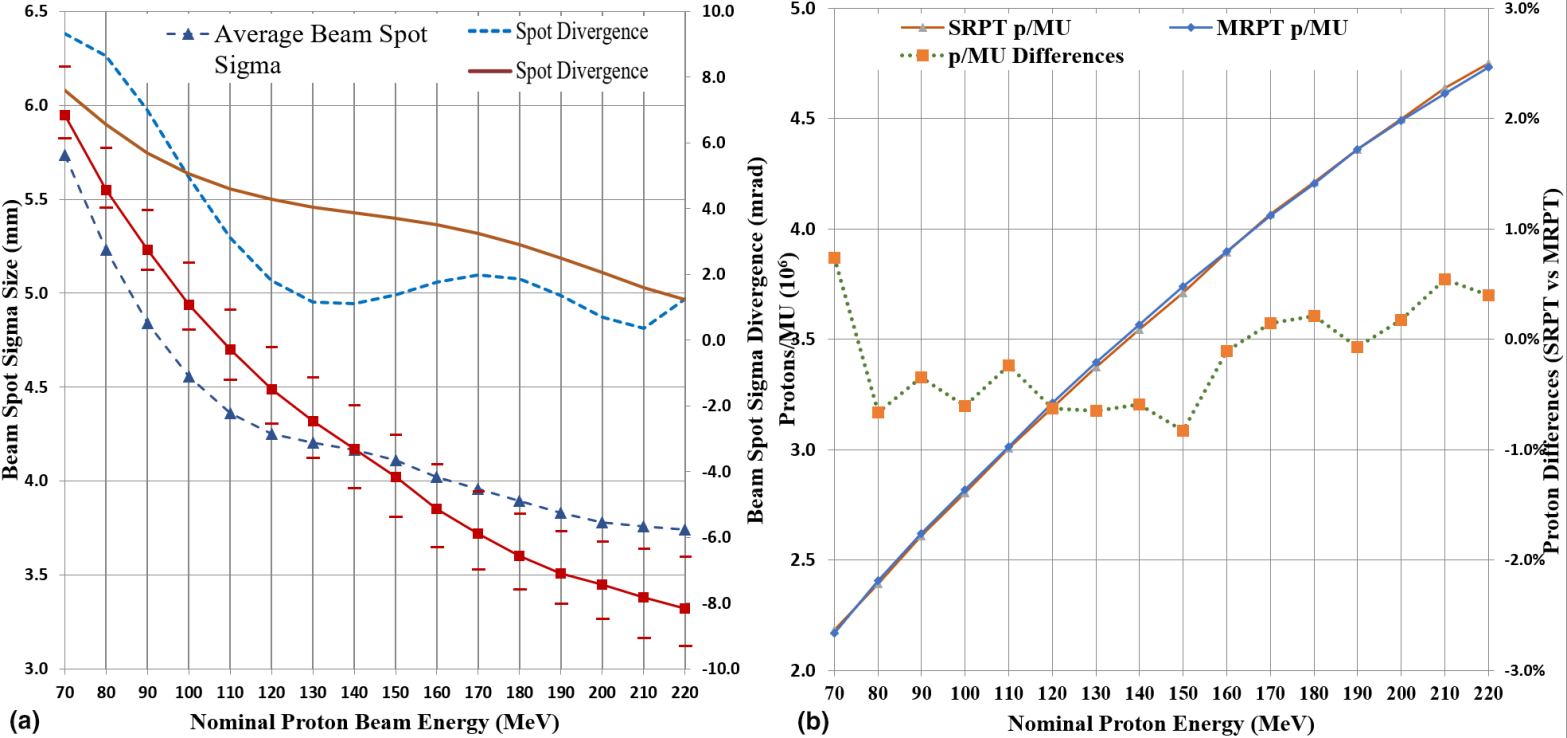
Proton beam spot comparison between single‐room ProBeam Compact™ proton therapy system (SRPT) (solid curves) and multi‐room ProBeam™ system (MRPT) (dotted curves) for (3a) average beam spot sigma at ISO, spot divergence; (3b) the number of protons/MU and their differences of SRPT vs MRPT.

Minor differences in spot sizes were identified between SRPT and MRPT as shown in Fig. 3(a), with spot divergences ranging within 7.62–1.23 mrad and 9.32–1.25 respectively. With similarly modeled AcurosPT algorithms, both institution Eclipse planning systems yield almost identical protons/MU for each nominal proton energy as shown in Fig. 3(b).

**Fig. 4:**
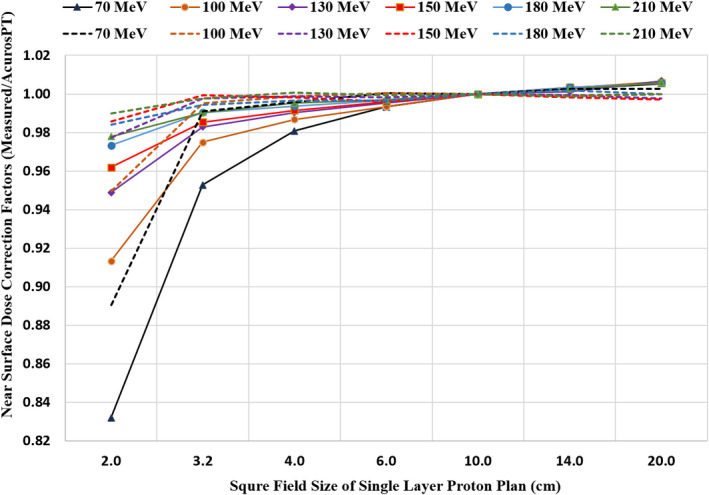
Comparisons of field size effects of single energy layer proton plans on the near surface (8 mm WET) doses, relative to 10 × 10 cm^2^ field doses, between those measured by a 0.07 cc ion chamber (solid curves) and computed by AcurosPT (dotted curves).

The halo effect from different field sizes of single layer proton plans is illustrated in Fig. 4 from the comparisons (Fig. 4) between the AcurosPT computed doses and the doses measured by Semiflex 0.07 cc (2.4 mm × 4.8 mm) pinpoint ion chamber. The latter was cross calibrated by the Octavius 1500ZDR 2D ion chamber array, the device dedicated for patient‐specific QAs. From the illustration, one can see that the largest correction factors or dose discrepancies are observed in lower energies with field sizes smaller than 4 × 4 cm^2^. In the 2 × 2 cm^2^ field‐sized 70 MeV proton plan, the AcurosPT model in current version overestimated the near surface dose up to +7.0% (as listed in Table 1), similar to the result of +6% published by Harms at al.^8^

**Table 1:** Near surface (WET = 8 mm) dose deviations between the computed by Eclipse AcurosPT and the measured by PTW Octavius ion chamber array andthe doses acquired by PTW Semilex 0.07 cc ion chamber using 70 MeV single‐layer proton beams at different field sizes.

Field size (cm)	AcurosPT	Octavius
2.0	−7.0%	0.4%
3.2	−4.0%	0.7%
4.0	−1.5%	0.8%
6.0	−0.7%	0.7%
10.0	0.0%	0.0%
14.0	0.0%	
20.0	0.3%	

Table 2 highlighted the reported results from two end‐to‐end independent checks by IROC on different phantoms show high passing scores. The two dosimetry checks are, respectively, for a uniform spread‐out Bragg peak plan using the IROC block phantom and an intensity‐modulated proton therapy (IMPT) prostate plan on a pelvic phantom.

**Table 2:** Results of IROC independent proton dose output and end‐to‐end treatment dosimetry checked on a pelvic phantom against Eclipse AcurosPT plans and delivery.

OSL point dose	IROC vs AcurosPT	Passing criteria
Uniform volumetric proton dose delivery to square phantom		
cGy at dose point	273: 273	
Ratio	1.00	0.95–1.05

TLD point dose	IROC vs AcurosPT	Criteria
Prostate IMPT to pelvic phantom		
Center prostate (L)	1.03	0.93–1.07
Center prostate (R)	1.04	0.93–1.07

Film plane dose	IROC vs AcurosPT	Criteria
Coronal	96%	≥85%
Sagittal	96%	≥85%

The analysis of patient‐specific QAs for over 108 proton fields, measured by Octavius‐1500 XDR ionization chamber 2D array, is summarized in Table 3, the average passing rates exceeded 95% and all comparisons pass 90% using Gamma index criteria of 3 mm, 3% (local), for fields with or without range shifters. All beams were measured in solid water at mid‐depth of SOPB and analyzed with PTW VeriSoft 7.20.68 software. In those collected initial plans, all target dimensions are >2.4 cm.

**Table 3:** Analysis of 108 patient‐specific individual field quality assurance (QA) Measurements.

Gamma index (GI)	Open field			Field w range shifter		
	# of QA	Pass rate	SD	# of QA	Pass rate	SD
3%, 3 mm	62	98.2%	2.3%	21	98.4%	2.6%
≤ 3%, 2 mm	11	96.3%	1.2%	14	95.9%	3.6%
>90% GI score		100.0%			100.0%	

## 4 DISCUSSION

The newly designed single gantry room ProBeam Compact™, SRPT met all the newly adapted beam specifications provided by the vendor.^19^ In comparing with the scanning proton beam data from a recent multi‐room ProBeam, SRPT exhibited a widened range straggling in the Bragg peaks and elevated proximal transmission beam dose, especially for the proton energies of 130 MeV or greater. This suggests that of the energy spectrum of the delivered proton beams for a given nominal proton energy is less confined, due to the challenges from the truncated beam transport system with inability of stripping off all the outlier energies from the energy selection assembly.^2^ When 250 MeV protons from the cyclotron pass through its energy selection system, the interactions of protons with the multi‐wedged attenuator create a narrow range of energy spectrum around the selected MeV which, after passing through a beam aperture, is sent to the beam transportation line on the gantry after. The energy spectrum of the proton beam will be further narrowed in the remaining beamline, particularly by the only two major bending magnets (45° at the beginning of the gantry and 135° on the top the gantry). In comparison, the additional bending magnets of MRPT already filter out some outliers of the beam spectrum, especially for more energetic proton beams, before redirected to the selected treatment vault. Thus, wider energy spectrum for selected energies of 130 MeV or higher constitute part of new beam characteristics of SRPT, although the similar properties and profiles of proton beam spots are still maintained.

In the current commissioning, the proton beam spot profiles are derived from single Gaussian fitting, partially ignored the minor dose deviations in the profile low dose tails or halo region. To overcome the modeling and algorithm deficiencies,^8^ an additional tuning for dose outputs was applied, particularly for AcurosPT model. Since AcurosPT is exclusively used as the final plan dose computing algorithm, a more careful output fine‐tuning was for improving the overall dosimetric accuracy within the scope of routine clinically used field sizes or 2.4 cm or greater in each dimension. With the similarly modeled Eclipse planning systems, the selected beam parameters included in the results are analytically comparable between SRPT and MRPT.

While the surface dose discrepancies increase more drastically with lower energy proton beams (Fig. 4), the majority of shallow dose is contributed by more consistent transmission doses of higher energy protons. Thus, in most of cases, the surface dose deviation could still be within the clinically acceptable range. Employment of a range shifter when applicable to avoid lowest energies can also further minimize the surface dose deviations for a shallow small target. As observed in Table 4, the surface dose disagreement for small fields (≤3.2°× 3.2 cm^2^) due to ignored halo created in the nozzle^8^ with our current beam model is reduced to below 1.4% by using a range shifter. For open field above 4.0°× 4.0 cm^2^, there is no clinically meaningful difference (≤1.5%) between measurement and AcurosPT for both open fields and fields with range shifter. The potential contributing factors may include measurement uncertainties and using different AcurosPT computation parameters, such as maximal simulation particles and resolutions.

**Table 4:** Near surface (WET = 8 mm) relative dose deviations between the computed by AcurosPT and the measured by PTW Semiflex 0.07 cc ion chamber using single‐layer beams of 70 MeV open proton field and 100 MeV proton beams with a 5 cmrange shifter (at 10 cm air gap) at different field sizes.

Field size (cm)	70 MeV open field			100 MeV with 5‐cm RS		
	Measured	AcurosPT	Deviation	Measured	AcurosPT	Deviation
2.0	0.83	0.89	−7.0%	0.67	0.66	1.4%
3.2	0.95	0.99	−4.0%	0.86	0.86	0.1%
4.0	0.98	1.00	−1.5%	0.93	0.94	−1.0%
6.0	0.99	1.00	−0.7%	0.96	0.97	−1.4%
10.0	1.00	1.00		1.00	1.00	

Realizing the limitations from our current beam modeling using single Gaussian instead of multiple Gaussians,^20^ one may implement some other work around, such as planed dose offset, to overcome computational dose deviations, especially for the cases with small target dimensions or with uncommon spot distributions. Optimally, more comprehensive beam modeling and spot fitting methodology will further improve the accuracy of proton dose computations with broader range of treatment conditions.^10^

## 5 CONCLUSIONS

In this submission, the author has highlighted the dosimetry differences in IDDs mainly introduced by a shortened beam transport system in the newly designed ProBeam Compact™, for which a set of new acceptance criteria was adapted. This report offers a unique reference for future commissioning, beam modeling, and planning on different systems, as well as analysis of dosimetric QA and clinical studies. With current beam modeling, a satisfactory planning quality and delivered dose accuracy are suggested by independent end‐to‐end tests and patient's specific beam QAs when mixed proton energies were used. However, beam modeling with more comprehensive methodology for all applicable planning algorithms is warranted for the future investigations.

## CONFLICT OF INTEREST

None.
